# Unidirectional spin-Hall and Rashba−Edelstein magnetoresistance in topological insulator-ferromagnet layer heterostructures

**DOI:** 10.1038/s41467-017-02491-3

**Published:** 2018-01-09

**Authors:** Yang Lv, James Kally, Delin Zhang, Joon Sue Lee, Mahdi Jamali, Nitin Samarth, Jian-Ping Wang

**Affiliations:** 1 Department of Electrical and Computer Engineering, University of Minnesota, Minneapolis, MN 55455 USA; 2 Department of Physics, The Pennsylvania State University, University Park, PA 16802 USA

## Abstract

The large spin−orbit coupling in topological insulators results in helical spin-textured Dirac surface states that are attractive for topological spintronics. These states generate an efficient spin−orbit torque on proximal magnetic moments. However, memory or logic spin devices based upon such switching require a non-optimal three-terminal geometry, with two terminals for the writing current and one for reading the state of the device. An alternative two-terminal device geometry is now possible by exploiting the recent discovery of the unidirectional spin Hall magnetoresistance in heavy metal/ferromagnet bilayers and unidirectional magnetoresistance in magnetic topological insulators. Here, we report the observation of such unidirectional magnetoresistance in a technologically relevant device geometry that combines a topological insulator with a conventional ferromagnetic metal. Our devices show a figure of merit (magnetoresistance per current density per total resistance) that is more than twice as large as the highest reported values in all-metal Ta/Co bilayers.

## Abstract (web summary)

Unidirectional spin Hall magnetoresistance enables the new spintronic devices but is limited by the low amplitude or working temperature. Here, the authors report the large unidirectional spin Hall magnetoresistance in a topological insulator and ferromagnetic metal bilayer system at relatively higher temperature.

## Introduction

The spin Hall effect (SHE) in non-magnetic (NM) heavy metals originates in their strong spin−orbit coupling (SOC) and has been extensively studied recently^1–4^. When a charge current flows through a NM heavy metal, the SHE yields a spin accumulation at the interface with a proximal material. If the latter is a ferromagnetic (FM) layer, the spin accumulation at the interface can exchange angular momentum with the magnetic moments and exert a spin-orbit torque (SOT). With certain configurations and sufficient charge current density, the magnetization in the FM can be switched. SOT switching is believed to be potentially faster and more efficient than spin transfer torque (STT) switching that is typically used in magnetic tunneling junction (MTJ) devices for memory and logic applications^3, 5, 6^.

SOT switching devices consist of a current carrying channel with a proximal nanomagnet whose magnetization determines the memory or logic state. Such devices need two terminals for writing the state of the device and an additional terminal, usually an MTJ on top of the nanomagnet, for reading the magnetization state of the device^3, 5^. Since the stable states of the nanomagnet are 180-degree-opposite to each other, symmetry prevents the sensing of the magnetization state using a conventional two-terminal magnetoresistance, such as anisotropic magnetoresistance or spin Hall magnetoresistance (SMR)^7, 8^. The required presence of a third terminal for reading makes such SOT switching devices more difficult or expensive to fabricate and usually less appealing for memory and logic applications.

With the recent discovery of unidirectional spin Hall magnetoresistance (USMR) in NM/FM bilayers, such as Pt/Co and Ta/Co, the third terminal of SOT switching devices is no longer necessary (Y. Lv and J.P. Wang, personal communication)^9–11^. USMR originates from the interactions between the spins generated at the NM/FM interface by SOC of the NM and the conduction channels in the FM. The unique feature of USMR is its symmetry; it is sensitive to two opposite magnetization states. Therefore, this allows one to envision a two-terminal SOT switching device that relies on USMR: the nanomagnet is switched by a current through the NM channel, while the state of the magnetization of the nanomagnet is simply read out using the USMR.

While much of the mainstream activities in SOT devices have focused on heavy metals, such as Ta, Pt, and W, recent research has begun to explore the potential of 3D topological insulators (TIs)^12–15^. These are narrow band gap semiconductors wherein strong SOC and time-reversal symmetry yield helical spin-textured Dirac surface states whose spin and momentum are orthogonal. This spin-momentum locking (SML) has been confirmed using direct measurements such as photoemission^13^, electrical transport^16–19^, and spin torque FM resonance^20^, as well as indirect means such as spin pumping^21–26^. It has also been demonstrated that the spins can exert torques on an FM^20, 27–29^ as one would expect of SOT in the NM/FM case.

In comparison to the NM/FM bilayers, where SOT switching and sensing using USMR have both been confirmed, the observation of USMR in TI/FM systems has not been reported yet. In a very recent study^30^, large unidirectional magnetoresistance (UMR), which behaves similarly as USMR in terms of longitudinal electrical properties has been observed in magnetic TIs; however, it is attributed to a different physical mechanism, namely the asymmetric scattering of electrons by magnons. This UMR is observed in Cr_*x*_(Bi_1−*y*_Sb_*y*_)_2−*x*_Te_3_/(Bi_1−*y*_Sb_*y*_)_2_Te_3_ heterostructures at very low temperatures (from 2 K up to 30 K). Although both USMR and UMR could play the same role of detecting magnetization in a two-terminal SOT switching device, the practical applicability of UMR in magnetic TIs is constrained by the low Curie temperature in these materials. Consequently, the UMR decays rapidly above 30 K in these systems. On the other hand, for more pragmatic applications, it is desirable to explore the USMR phenomenon in heterostructures that interface a TI with a conventional FM of technological relevance.

**Fig. 1:**
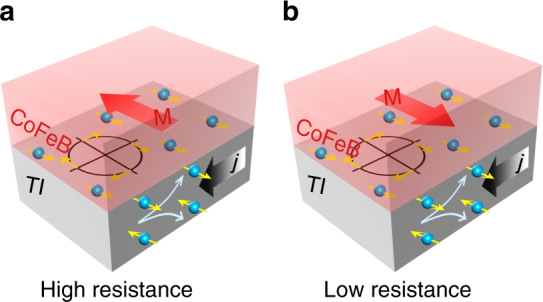
Illustration of USRMR in TI/FM bilayer. Spin accumulation is generated at the interface and in the bulk when a charge current is applied. The relative direction of the spin polarization to the magnetization of either **a** parallel and **b** anti-parallel results in different resistance states

In this work, we report the experimental observation of USMR-like magnetoresistance in TI/FM heterostructures, including (Bi,Sb)_2_Te_3_/CoFeB and Bi_2_Se_3_/CoFeB bilayers. Our analysis of the data indicates that the USMR picture provides a more appropriate description of our observations. However, we also determine that the observed magnetoresistance originates from both bulk and surface contributions of spin-charge conversion in the TI films. Therefore, to better reflect the underlying mechanisms, we refer to the observed UMR as unidirectional spin-Hall and Rashba−Edelstein magnetoresistance (USRMR). As illustrated in Fig. 1, spins are generated due to the SML of the TI as well as the strong SOC in the bulk of the TI when a charge current, *j*, is applied in the bilayer. Depending on the relative directions between the spins and magnetization of FM, spins at the interface present different conductance when interacting with the conduction channels in the FM. The USRMR in TI/FM systems is like that in NM/FM systems but with different mechanisms of spin generation.

## Results

We observe USRMR at temperatures between 20 and 150 K for (Bi,Sb)_2_Te_3_ (BST) and Bi_2_Se_3_ (BS). The largest USRMR among our samples is more than twice as large as the best USMR in Ta/Co samples, in terms of USRMR (or USMR) per total resistance per current density. This value is observed in a six quintuple layer (QL) BS and 5-nm-thick CoFeB bilayer at 150 K. The devices studied are fabricated from BST (*t* QL)/CoFeB (5 nm)/MgO (2 nm) and BS (*t* QL)/CoFeB (5 nm)/MgO (2 nm) thin film stacks (*t* = 6 and 10), grown by molecular beam epitaxy (MBE) and magnetron sputtering. Hall bars of 50 μm in length and 20 μm in width are tested with harmonic measurements under both longitudinal and transverse resistance measurement setup. The magnetization of CoFeB is spontaneously in-plane with little perpendicular anisotropy field.

### First and second harmonic resistance angular dependence

**Fig. 2:**
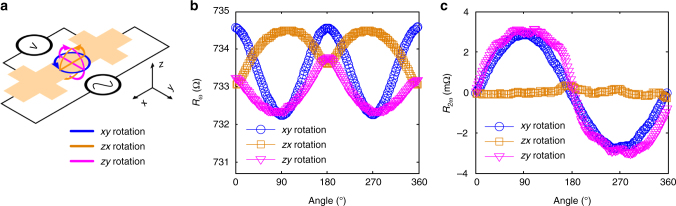
Longitudinal resistance. **a** Longitudinal resistance measurement setup and definitions of rotation planes. **b** First harmonic and **c** second harmonic resistances of 10 QL BST sample at 150 K are shown when the external field is rotated in three orthogonal planes. The starting points and zero angles are at *x*+, *y*+, and *z*+, the directions of rotation for increasing angle are *x* to *y*, *z* to *x,* and *z* to *y*, for *xy*, *zx,* and *zy* rotations, respectively

Figure 2a shows the definition of the coordinates and rotation planes. Zero angles are at *x*+, *y*+ and *z*+ directions for *xy*, *zx*, and *zy* rotations respectively. The directions of rotation for increasing angle are indicated by the arrows. A 3 T external field is applied and rotated in the *xy*, *zx*, and *zy* device planes while the first harmonic resistance *R*_*ω*_ and second harmonic resistance *R*_2*ω*_ are recorded with 2 mA RMS A.C. current. Figure 2b and c shows the angle dependencies of *R*_*ω*_ and *R*_2*ω*_, respectively, of the BST (10 QL)/CoFeB (5 nm)/MgO (2 nm) sample at 150 K. The *R*_*ω*_ exhibits typical SMR-like behavior with *R*^*x*^ > *R*^*z*^ > *R*^*y*^. Similar to the behavior seen in all metallic NM/FM bilayers, the variation of the second harmonic resistance *R*_2*ω*_ with angle is also proportional to the magnetization projected along the *y*-direction. The period of the *xy* and *zy* rotations is 360 degrees, while a flat line is observed in the *zx* rotation. The amplitude of *R*_2*ω*_ is about 3 mΩ with an average current density of 0.667 mA cm^−2^.

### Extraction of USRMR

**Fig. 3:**
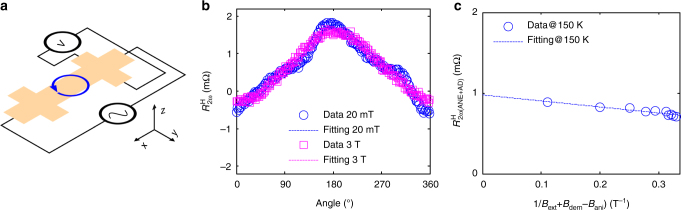
Hall resistance. **a** Hall/transverse resistance measurement setup. **b** Examples of second harmonic Hall resistance of 10 QL BST sample at 150 K vs angle in *xy* plane rotation with 20 mT and 3 T external fields. **c** Hall resistance measured with various external fields is plotted vs reciprocal of total field. The dashed line is a linear fit to the data; the intercept of the fitted line represents the contribution of ANE/SSE

The heat generated by Joule heating of the device film dissipates mostly vertically across the film plane and creates a temperature gradient. This temperature gradient gives rise to the anomalous Nernst effect (ANE) and spin Seebeck effect (SSE). The ANE is a thermoelectric-effect-driven-version of the anomalous Hall effect (AHE) of an FM, which originates from SOC in an FM^31^. The SSE, on the other hand, is the result of the conversion of spin current injected by the thermoelectric effect into charge current in the NM^32^. Regardless, both ANE and SSE behave similarly in our bilayer system as well as in other FM/NM systems; their total contribution to the electric field or voltage is proportional to *j*^2^**M** × **∇***T*, where **M** is magnetization and **∇***T* is temperature gradient. The factor *j*^2^ indicates that the voltage associated with ANE/SSE is of second order. Also, we note that the ANE/SSE both contribute to the longitudinal second harmonic signal when **M** is along the *y*-direction and to the transverse signal when **M** is along the *x*-direction. To carefully separate this contribution (denoted as $R_{2{\omega }}^{{\mathrm{\Delta T}}}$) from the measured *R*_2*ω*_ to verify USRMR, we carried out a series of measurements of Hall or transverse second harmonic resistance with *xy*-plane rotations under various external field strengths. Figure 3a shows the Hall resistance setup. The transverse resistance is measured while the external field is rotated in the *xy*-plane. The second harmonic Hall resistance, $R_{2{\omega }}^{\mathrm{H}}$, contains contributions from ANE/SSE, field-like (FL) SOT and anti-damping (AD) SOT. The ANE/SSE and AD SOT are proportional to $\cos{\varphi }$ while the FL SOT is proportional to cos3*φ* + cos*φ*^33^. Figure 3b shows two examples of $R_{2{\omega }}^{\mathrm{H}}$ vs angle with 20 mT and 3 T external fields, respectively. Since the AD SOT and the FL SOT perturb the magnetization and thus contribute to $R_{2{\mathrm{\omega }}}^{\mathrm{H}}$ through the AHE and the planar Hall effect, their effects diminish at larger external field. Figure 3b also shows that the data measured under a 20 mT field contain both cosφ and cos3*φ* components, while under a 3 T field, the data exhibit almost no cos3*φ* component. There are two steps to obtain the $R_{2{\omega }}^{{\mathrm{\Delta T}}}$. First, by fitting the angle-dependent data, we extract the amplitudes of the cos*φ* and cos3*φ* components. The FL SOT can then be easily determined and separated. This leaves the contributions of the ANE/SSE and the AD SOT, $R_{2{\omega }({\mathrm{ANE}} + {\mathrm{AD}})}^{\mathrm{H}}$, to the measured total Hall signal, $R_{2{\omega }}^{\mathrm{H}}$. We plot the data corresponding to these contributions vs the reciprocal of total field, as shown in Fig. 3c. In this figure, *B*_dem_−*B*_ani_ is the demagnetization field minus the perpendicular anisotropic field of the FM layer, which is determined to be about 1.5 T by separate AHE measurements (see Supplementary Note 1 and Supplementary Fig. 1). Since the effect of the AD SOT will diminish at infinite field, the intercept of the fitted line is the contribution of ANE/SSE to the second harmonic Hall resistance. Then, we can obtain the contribution of ANE/SSE to the longitudinal resistance *R*_2*ω*_ by scaling that from the Hall resistance with the relative ratio of device length to device width. Finally, the USRMR is determined once the ANE/SSE contribution is subtracted from the *R*_2*ω*_. Note that the suppression of the SSE by high external field does not invalidate the assumption of the ANE/SSE signal being constant since the suppression is only reported in thick Pt/YIG systems (with YIG thicker than 310 nm)^34^. There is no report on high-field suppression of the SSE in CoFeB yet. Further, our measurements of *R*_2*ω*_ vs *H*_*y*_ confirm the absence of a change in amplitude of the SSE up to 3 T field in BS (10 QL)/CoFeB (5) bilayer (see Supplementary Notes 2–4 and Supplementary Figs. 2–4) and of the ANE up to 7 T field in 5-nm single-layer CoFeB (see Supplementary Note 2 and Supplementary Fig. 2).

### Second harmonic resistance components

**Fig. 4:**
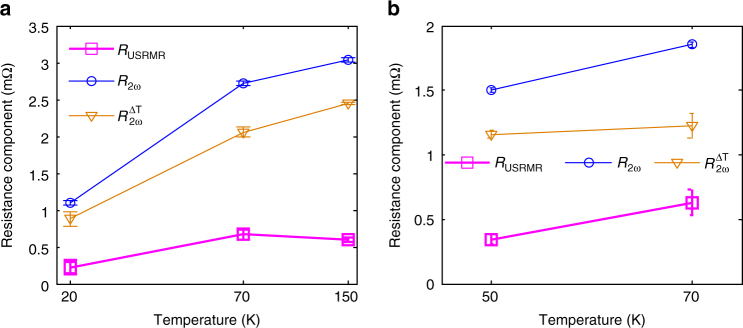
Second harmonic resistance components. The measured second harmonic longitudinal resistance, *R*_2*ω*_, consists of contribution of $R_{2{\omega }}^{{\mathrm{\Delta T}}}$ and USRMR, *R*_USRMR_. Each component is plotted vs temperature for **a** 10 QL BST sample and **b** 10 QL BS sample. The error bars indicate uncertainty bounds with 95% confidence. The uncertainties reflect the variations of observed signal level in the field sweep and angle rotation data (see Supplementary Note 8 for details)

Figure 4a and b shows the *R*_2*ω*_, $R_{2{\omega }}^{\Delta {\mathrm{T}}}$, and *R*_USRMR_ of 10 QL BST (Fig. 4a) and 10 QL BS (Fig. 4b) samples with 2 and 3 mA currents, respectively, at various temperatures. Here, the error bars indicate uncertainty bounds with 95% confidence (see Supplementary Note 8). Temperature affects the chemical potential and the relative contributions to transport from surface and bulk conduction in TIs. As a result, even though the magnetization and resistivity of the CoFeB layer vary little within the range of temperature in our experiments (see Supplementary Note 5 and Supplementary Fig. 5), the charge to spin conversion in TIs and the related USRMR are both temperature dependent. The 10 QL BST/CoFeB sample gives its highest USRMR at 70 K, while the *R*_2*ω*_ and $R_{2{\omega }}^{{\mathrm{\Delta T}}}$ keep increasing with increasing temperature up to 150 K. The USRMR of 10 QL BS/CoFeB can only be confirmed within between 50 and 70 K because of larger noise and magnetic-field-dependent signal outside this temperature window (see Supplementary Note 7 and Supplementary Figs. 7–9 for more information). At 70 K, BST and BS samples show resistance *R*^*z*^ of 733 Ω and 488 Ω, and USRMR per current density of 1.00 ± 0.11 and 0.63 ± 0.10 mΩ MA^−1^ cm^2^, respectively. The ratios of USRMR per current density to total resistance of the two samples are 1.37 ± 0.15 and 1.29 ± 0.20 ppm MA^−1^ cm^2^, respectively. These values are slightly better than the best result obtained using Ta/Co bilayers (1.14 ppm MA^−1^ cm^2^ at room temperature)^9^.

### USRMR figure of merit and sample comparisons

**Fig. 5:**
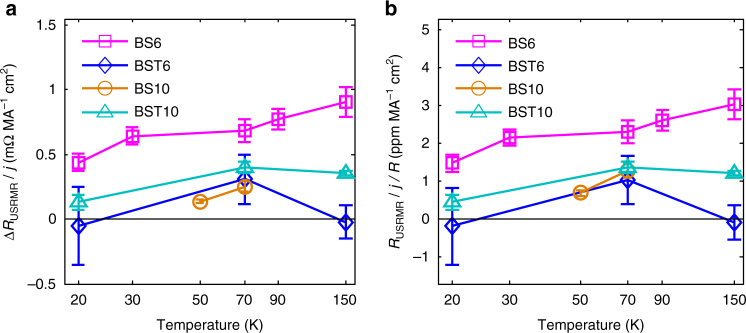
Summary of USRMR. **a** Sheet USRMR per current density and **b** USRMR per current density per total resistance of all four samples at various temperatures. BS{*x*} or BST{*x*} are abbreviations of BS or BST samples of {*x*} QL thicknesses. The error bars indicate uncertainty bounds with 95% confidence. The uncertainties reflect the variations of observed signal level in the field sweep and angle rotation data (see Supplementary Note 8 for details)

Since the USRMR is a second harmonic nonlinear resistance, its amplitude is proportional to current or current density. For each different material system of various thicknesses tested with different Hall bar dimensions, normalizing the USRMR over current density helps to eliminate variations in the results caused by specific testing current, Hall bar dimensions, and thin film stack thicknesses. On the other hand, we can conveniently estimate the maximum USRMR possible for each system by using current density instead of total current to normalize the USRMR. This is because the limiting factors, namely, Joule heating and device break down, are more closely related to current density. Furthermore, in a potential application scenario, where bilayer sandwich thin film stacks are patterned into rectangles to make SOT switching devices and the current is driven in the film plane for both SOT magnetization switching and USRMR magnetization detection, the amplitude of the USRMR, as well as the total resistance, of this kind of device is only dependent on the lateral aspect ratio as the devices scale. Therefore, we propose using sheet USRMR, which is measured as USRMR divided by the aspect ratio of Hall bar, per current density (Δ*R*_USRMR_*/j*) as a possible figure of merit for comparisons of USRMR across diverse types of material systems. This value indicates the amplitude of USRMR normalized by (and regardless of) specific device dimensions and testing conditions. We now consider two material systems providing the same Δ*R*_USRMR_*/j*, but of very different resistivity or total resistance *R*. When measuring the USRMR of the one with larger *R*, the instruments or circuitry must isolate the nonlinear signal corresponding to the USRMR from the larger linear background caused by *R*. Clearly, the one with lower *R* is more desirable for detecting the USRMR. Thus, we propose another figure of merit, *R*_USRMR_*/j/R*, to indicate the relative signal change or amplitude of the USRMR, that is also normalized by specific device dimensions and testing conditions. Note that *R*_USRMR_*/j/R* *=* Δ*R*_USRMR_*/j/*Δ*R*. Figure 5 shows the sheet USRMR per current density, Δ*R*_USRMR_*/j*, (a) and the USRMR per current density per total resistance, *R*_USRMR_*/j/R*, (b) for all four samples as a function of temperature (BS{*x*} or BST{*x*} are abbreviations of BS or BST samples of {*x*} QL thicknesses); the error bars indicate uncertainty bounds with 95% confidence (see Supplementary Note 8). These two values also show very similar trends for all samples at various temperatures, except for the comparison between BST6 and BS10 at 70 K, in which BST6 is lower than BS10 in terms of *R*_USRMR_/*j*/*R* but higher than BS10 in terms of Δ*R*_USRMR_*/j*. The swap of position is mostly due to the larger total resistance of BST6 compared to BS10, while they show comparable *R*_USRMR_*/j*. We could also see that at 20 and 150 K, the BST6 does not show greater-than-zero USRMR reasonably beyond the confidence of the measurement. The largest values of Δ*R*_USRMR_*/j* and *R*_USRMR_*/j/R* are 0.90 ± 0.12 mΩ MA^−1^ cm^2^ and 3.05 ± 0.39 ppm MA^−1^ cm^2^, respectively, and both are observed in BS6 at 150 K. These values are more than twice as large as the best reported in the Ta/Co case^9^. The USRMR measurements beyond the temperature ranges of the plots of each sample show strong noise and field-dependent signal background as to render the estimations of USRMR unreliable (see Supplementary Note 7 and Supplementary Figs. 7–9 for more information).

As previously mentioned, the magnetization and resistivity of CoFeB vary only by less than 10% throughout the temperature range of our experiments (see Supplementary Note 5 and Supplementary Fig. 5). However, the USRMR, as shown in Fig. 5, varies significantly at various temperatures and between samples. We believe that the amount of current flowing in TIs dictates the USRMR performance of each sample at various temperatures. The basic transport properties of single-layer same-batch TI samples are summarized in Table 1. The resistivity, *ρ*_*xx*_, of TIs is about as low as 5 times (BS6) to as high as 42 times of that of a 5-nm single-layer CoFeB. This major difference of resistivity between the TI layer and the CoFeB layer leads to a small fraction of the charge current flowing in the former, which is then converted to spin accumulation at the interface and results in USRMR. When comparing the USRMR at 70 K across all four samples, we notice that the BS6 exhibits the highest USRMR while being the least resistive and of the largest carrier concentration (average), *n*_3D_. Another observation is that the BST6 sample has very low sheet carrier concentration down to 1.14×10^12^ range. This sample, based on its *n*_3D_, resistivity, and mobility, is almost an ideal TI. But surprisingly, this sample does not yield high USRMR. And in contrast, the BST10, which has *n*_3D_ of an order of magnitude higher, which is considered having considerable bulk conduction, exhibits higher USRMR. Then considering again, among the two BS samples, the one with higher *n*_3D_ also yields higher USRMR, we believe that it is very likely that in TI/FM systems, due to the large resistivity mismatch between ideal TI and FM, appropriate amount of bulk conduction on TI could help improving USRMR performance. And with this hypothesis, the overall trend of USRMR in sample BS6 decreased with lower temperature can be interpreted as the freezing of bulk conduction of TI. But this might only be significant when transitioning between a high temperature (70−150 K, for our experiments) to a very low temperature (20 K, for our experiments), where the freezing of bulk carriers is signification.

**Table 1:** Summary of transport properties of bare TI samples

Sample	BS6	BS10	BST6	BST10
Temperature (K)	2.0	1.8	2.5	4.2
*ρ*_*xx*_ (mΩ cm)	0.636	0.765	4.50	5.86
*μ* (cm^2^ V^−1^ s^−1^)	246	474	730	58.8
Type	n-type	n-type	p-type	p-type
*n*_2D_ (cm^−2^)	2.40×10^13^	1.72×10^13^	1.14×10^12^	1.81×10^13^
*n*_3D_ (cm^−3^)	3.99×10^19^	1.72×10^19^	1.90×10^18^	1.81×10^19^

Bare TI samples made in the same batches with the BS{6, 10} and BST{6, 10} for USRMR study are referred in this table with the same names, but they are bare without CoFeB deposition. The resistivity, *ρ*_xx_, and sheet carrier concentration, *n*_2D_, are measured from Hall bars of 1 mm in length and 0.5 mm width. Then the mobility, *μ*, and average carrier concertation, *n*_3D_, are calculated

## Discussion

In summary, we demonstrated the presence of USRMR in a new material system category: TI/FM layer heterostructures. The USRMR was observable with a much lower current density compared to all metallic NM/FM bilayers. The ratios of the USRMR per current density to total resistance are found to be comparable to the best result reported so far in Ta/Co bilayers. The USRMR performance is believed to be heavily dependent on specific TI transport properties and conditions. The observation of USRMR in a TI/FM system is an important part of the puzzle to build a two-terminal TI-based SOT switching device. Such a two-terminal topological spintronic switching device is potentially more efficient compared to MTJs that use STT switching due to the large SOC of TIs. The USRMR we observe could enable the read operation of such a device without having to build an MTJ structure on top of the TI. Such two-terminal devices are much more architecture friendly and more readily embedded in current STT magnetic random-access memory architectures.

## Methods

### Thin film sample preparation

The Bi_2_Se_3_ or (Bi_1−*x*_Sb_*x*_)_2_Te_3_ films were grown by MBE on semi-insulating InP (111) substrates. The InP (111) substrate is initially desorbed at 450 °C in an EPI (Veeco) 930 MBE under high purity (7N) As_4_ supplied by a Knudsen cell until a 2×2 reconstruction is visible in reflection high-energy electron diffraction. The substrate is then moved under vacuum to an EPI 620 MBE for the Bi-chalcogenide deposition. Bi_2_Se_3_ films were grown from high purity (5N) Bi and Se evaporated from Knudsen cells at a beam equivalent pressure flux ratio of 1:14. The substrate temperature was 325 °C (pyrometer reading of 250 °C) and the growth rate was 0.17 nm min^−1^. The 6 QL Bi_2_Se_3_ film has a root mean squared (RMS) roughness of approximately 1.55 nm over a 25 μm^2^ area measured by atomic force microscopy (AFM). For (Bi,Sb)_2_Te_3_ films, the flux ratio of Bi to Sb was 1:3 and (Bi + Sb):Te is at a flux ratio of approximately 1:12 for a growth rate of 0.44 nm min^−1^. The 6 QL (Bi,Sb)_2_Te_3_ film has an RMS roughness of approximately 1.98 nm over a 25 μm^2^ area measured by AFM. These films are grown at a substrate temperature of 315 °C (240 °C measured by a pyrometer) using 5N purity Sb and 6N Te from Knudsen cells. Film thickness is measured by X-ray reflectivity and crystal quality by high-resolution X-ray diffraction rocking curves of the (006) crystal plane—with a full width half max of approximately 0.23 and 0.21 degrees for the 6 QL Bi_2_Se_3_ and (Bi,Sb)_2_Te_3_ films, respectively.

The MBE-grown TIs were then sealed in argon gas and transported to an ultra-high vacuum six-target Shamrock sputtering system which could achieve a base pressure better than 5 × 10^−8^ Torr at room temperature. The thin films were first gently etched by argon ion milling (see Supplementary Note 6 and Supplementary Fig. 6 for impact of such etching on TI). Then the CoFeB layer was deposited using a Co_20_Fe_60_B_20_ target. Finally, an MgO layer was deposited to serve as a protection layer.

### Device fabrication

The device fabrication began with photolithography followed by ion milling etching to define the Hall bars. Then, we carried out a second photolithography step and an e-beam evaporation, followed by liftoff to make contacts.

### Electrical measurements

The devices were tested in a Quantum Design PPMS that provides temperature control, an external magnetic field, and sample rotation. The AC current at 10 Hz was supplied by a Keithley 6221 current source. A Stanford Research SR830 or an EG&G 7265 lock-in amplifier paired with an EG&G 7260 lock-in amplifier were used to measure the first and second harmonic voltages, respectively and simultaneously.

### Data availability

The data that support the findings of this study are available from the authors on reasonable request; see author contributions for specific data sets.

## Electronic supplementary material

Supplementary Information
