# Supplementary material for: Unidirectional spin-Hall and Rashba−Edelstein magnetoresistance in topological insulator-ferromagnet layer heterostructures
Source: Nat Commun. 2018 Jan 9;9:111. doi: 10.1038/s41467-017-02491-3 (PMC5760711; doi:10.1038/s41467-017-02491-3)
Supplement: Supplementary file 1 — Supplementary Information [file 41467_2017_2491_MOESM1_ESM.docx]

**Supplementary Note 1. | Anomalous Hall effect of single-layer CoFeB (with MgO capping).** A 5-nm CoFeB layer followed by a 2-nm MgO capping layer is deposited on SiO_2_/Si substrate (Note that this thin film stack is referred as *5-nm CoFeB sample* without mentioning the MgO capping layer in the rest of this supplementary information). Then the stack is patterned into Hall bars. By measuring the anomalous Hall effect while sweeping the external field in the perpendicular direction, the *B*_dem_-*B*_ani_ is determined to be 1.5 T. As shown in **Supplementary Figure 1**, the *R*^H^ signal saturates at 1.5 T perpendicular field, *H*_z_.

**Supplementary Figure 1. |** **Hall resistance, *R*^H^, vs. the applied perpendicular field, *H*_z_, of the 5-nm CoFeB sample.** The Hall bar is of 30 μm length and 20 μm width and measured with 0.1 mA D.C. current at 300 K.

**Supplementary Note 2. | Anomalous Nernst effect of the 5-nm CoFeB sample at high external fields.** The same Hall bar device is measured with the harmonic measurement setup described in main text **Methods** section. As shown in **Supplementary Figure 2**, the *R*_2ω_ signal, which originates from the anomalous Nernst effect (ANE), remains in constant amplitude up to 7 T external field that is applied in the plane of the sample and transverse to the charge current direction *x*.

**Supplementary Figure 2. | Second harmonic resistance, *R*_2ω_, vs. in-plane transverse field, *H*_y_, of the 5-nm CoFeB sample.** The Hall bar is of 30 μm length and 20 μm width and measured with 2 mA A.C. current at 300 K.

**Supplementary Note 3.** **| Magnetization of the 5-nm CoFeB sample at high external fields.** The 5-nm CoFeB sample is measured using a vibrating sample magnetometer (VSM), which is a module of the PPMS^®^ DynaCool^TM^ manufactured by Quantum Design, Inc. As shown in **Supplementary Figure 3,** the magnetization saturates quickly at low field and remains stable up to 8 T.

**Supplementary Figure 3. | Magnetization, *M*, of the 5-nm CoFeB sample vs. in-plane field,** $\boldsymbol{H}_{\mathbf{in-plane}}$**.** The magnetization of the 5-nm CoFeB sample is measured using a vibrating sample magnetometer while an in-plane external field is swept. The inset shows a zoom of *M* around 1300 emu cm^-3^ with *H*_in-plane_ >0. **Supplementary Note 4. | Contributions of ANE and spin Seebeck effect to *R*_2ω_ at high external fields.** The measured *R*_2ω_ signal contains unidirectional spin-Hall and Rashba-Edelstein magnetoresistance (USRMR) along with the contributions from the ANE and the spin Seebeck effect (SSE). The estimation of the USRMR involves the assumption that the contributions from the ANE and the SSE to *R*_2ω_ remain constant up to high external field^1^. The control exeriment data in **Supplementary Figures 2 and 3** and **Supplementary Notes 2 and 3** shows that the magnetization and ANE of the 5-nm CoFeB sample saturates quickly with low field and remains constant in amplitude up to 7 T. In addition, **Supplementary Figure 4** shows that the *R*_2ω_ signal does not visibly change in its amplitude with an applied external magnetic field up to 3 T. This evidence supports the above assumption that both the ANE and the SSE remain constant in amplitude under high external field. We note that studies of Pt/YIG bilayers have shown a suppression of the SSE under a high magnetic field; this was attributed to the suppression of low-frequency magnons by the high magnetic field^2^. However, such suppression quickly becomes negligible when the thickness of the YIG approaches 310 nm. In contrast, the SSE in bilayers containing 5-nm CoFeB and TIs may only involve magnons of higher frequencies, which are not perturbed by an external field of a few Tesla.

**Supplementary Figure 4. | Second harmonic resistance, *R*_2ω_, vs. the in-plane transverse field *H*_y_, of BST10 sample at 150 K.** The BST10 sample is described in the main text being of 10 QL BST/ 5 nm CoFeB/2 nm MgO. The Hall bar is 30 μm in length and 20 μm in width and is measured with an A.C. current of 2 mA at 300 K.**Supplementary Note 5. | Saturation magnetization (a) and electrical resistivity (b) of the 5-nm CoFeB sample.** The saturation magnetization, *M*_S_, vs. temperature, as shown in **Supplementary Figure 5 (a)**, is extracted from the magnetization vs. applied magnetic field data measured by VSM described in **Supplementary Note 3**. As temperature decreases from room temperature to 10 K, the *M*_S_ increases from 1240 emu cm^-3^ to 1320 emu cm^-3^. The **Supplementary Figure 5 (b)** shows that the resistivity of CoFeB decreases from 142 μΩ cm to 139 μΩ cm when the temperature decreases from 300 K to 10 K. The resistivity is measured with the patterned Hall bar, as described in **Supplementary Note 1**, nominal thickness, width, and length are used to calculate the resistivity.

**(a)**

**(b)**

**Supplementary Figure 5. | Saturation magnetization, *M*_S_, (a) and electrical resistivity, *ρ*_xx_, (b) of the 5-nm CoFeB sample. Supplementary Note 6. | Impact of Argon ion mill etching on TI transport property.** For the TI/FM samples used in our USRMR study, before depositing CoFeB, we use an *in-situ* Argon ion milling with a low power of 10.5 W for 30 seconds to remove any potential contamination and oxidation of the TI samples during handling. For a control measurement of the effect from Ar ion milling on the TI samples, a new sample of 10 QL BS was grown. The sample was cleaved into two pieces, one to be etched by the Ar ion milling, while the other is the unetched control. Then, the samples are patterned into Hall bars and measured for electrical resistivity. During fabrication, the two sample pieces are handled side-by-side as much as possible to ensure a similar amount of oxygen exposure. During the ion milling, both samples are loaded into our deposition tool under vacuum with one of the samples etched by the ion milling. As shown in **Supplementary Figure 6**, the resistivity of the two samples vs. temperature trend very closely. Therefore, we believe that the low-power ion milling does not significantly change transport properties. Note, that this batch of 10 QL BS is grown in our molecular beam epitaxy tool at a later time and happens to be a more ideal TI, based on the values and the profile of the *ρ*_xx_ vs. temperature curve.

**Supplementary Figure 6. | Resistivity, *ρ*_xx_, vs. temperature of Argon ion mill etched and unetched 10 QL BS samples.** The blue line with circles indicates the *ρ*_xx_ of the sample that was not etched by ion mill etching while the red line with squares indicates *ρ*_xx_ of the etched sample. Each sample was measured with a D.C. current of 10 μA in a Hall bar geometry.

**Supplementary Note 7. | USRMR estimations of BST6 and BS10 at other temperatures.** For sample BST6 and BS10, we have additional datasets at lower and higher temperatures other than what is shown in the main text fig. 5(a). We estimated the USRMR and show the sheet USRMR per current density in **Supplementary Figure 7**. The additional points are plotted with thinner lines for error bar and dashed lines connecting them with the original data points. We do not show these additional points in the main text because we believe the estimations are likely unreliable based on the noisy or strange features in the raw data.

For the sample BST6, the USRMR at 50 K is deemed unreliable due to the following two symptoms. Firstly, the *R*_2ω_ signal vs. *H*_y_, as shown in **Supplementary Figure 8 (a)** (red line), does not saturate at higher magnetic fields, compared to the *R*_2ω_ measured at 70 K (blue line) which saturates. The 50 K data shows two obvious dips around -1.5 T and 2.4 T. Secondly, the $R_{2\omega}^{H}$ signal shown in **Supplementary Figure 8 (b)** (two example data), which contributes the most error to the estimation of USRMR, shows unstable readings and spikes. These two symptoms indicate possible electrical contact instability at 50 K. And this instability might be related to the extremely low carrier concentration in sample BST6 and its roughness.

For the 30 K point of the sample BS10, its $R_{2\omega}^{H}$ vs. angle plot, shown in **Supplementary Figure 9 (a)**, exhibits an antisymmetric component, which is neither $cos\varphi$ nor $cos3\varphi$. It is possible that there was mixing between the longitudinal and transverse signals. As a result, the USRMR is below zero, which we deem unreliable. The *R*_2ω_ signal vs. *H*_y_ at 100 K, shown in **Supplementary Figure 9 (b)**, sits on a large linear background. The step part of the signal corresponds to the magnetization switching while the linear background is magnetic field dependent. Such large field-dependent background possibly originates in the ordinary Hall effect in the semiconducting TI layer. The presence of such a large background could lead to other secondary effects on the amplitude of *R*_2ω_ associated with magnetization switching. Therefore, to be cautious we deem this point unreliable. Note that for the BS10 sample, we also attempt measurements at 150 K and collected *R*_2ω_ vs. *H*_y_ data. However, the field-dependent background is even larger.

**Supplementary Figure 7. | Sheet USRMR per current density of all four samples at various temperatures.** The plot is similar to fig. 5(a) in the main text with additional USRMR estimations of BST6 at 20, 50 and 150 K and BS10 at 30 and 100 K. These additional data points are represented by error bars with thinner lines and are connected by dashed lines.

**(a)**

**(b)**

**Supplementary Figure 8. | The BST6 sample’s (a) second harmonic longitudinal resistance, *R*_2ω_, vs. in-plane transverse field, *H*_y_, and (b) second harmonic Hall resistance,** $\boldsymbol{R}_{\boldsymbol{2\omega}}^{\mathbf{H}}$ **vs. angle in *xy*-plane rotation.** (a) is a direct plot of the raw data with *R*_2ω_ between -30 mT and 30 mT omitted because the lock-in amplifier was transitioning from the large change of *R*_2ω_ signal around 0 T. (b) The $R_{2\omega}^{H}$ data uses a low-pass filter with a moving-average filter spanning 2^o^ to better show the features of issue.

**(a)**

**(b)**

**Supplementary Figure 9. | The BST10 sample’s (a) second harmonic Hall resistance,** $\boldsymbol{R}_{\boldsymbol{2\omega}}^{\mathbf{H}}$ **vs. angle in *xy*-plane rotation at 30 K and (b) second harmonic longitudinal resistance, *R*_2ω_, vs. in-plane transverse field, *H*_y_, at 100 K.** The $R_{2\omega}^{H}$ signal in (a) is low-pass filtered with a moving-average filter spanning 2^o^ to better show the features of issue. (b) Is a direct plot of raw data with data between -30 mT and 30 mT omitted since the lock-in amplifier was transitioning from the change of $R_{2\omega}^{H}$ around 0 T.

**Supplementary Note 8. | Error estimation of measured *R*_2ω_,** $\boldsymbol{R}_{\boldsymbol{2\omega}}^{\mathbf{H}}$**, and calculated** $\boldsymbol{R}_{\boldsymbol{2\omega}}^{\boldsymbol{\Delta T}}$ **and *R*_USRMR_.** The uncertainties in *R*_2ω_ and $R_{2\omega}^{H}$ consist mainly of a low-frequency variation and high-frequency noise. While the lock-in amplifier’s filtering circuit/algorithm can filter the high-frequency noise, the low-frequency variation is not filtered effectively. In the second harmonic voltage readings the low-frequency variation is approximately 1~2 μV. We believe this low-frequency variation originates from the instability in the phase difference between the A.C. current source and the two lock-in amplifiers. In order to minimize the impact of this low-frequency variation when measuring *R*_2ω_ and $R_{2\omega}^{H}$, we stay at/around each applied magnetic field or rotation angle long enough to average out the low frequency variation in the resistance signals. The data is then analyzed using nonlinear least squares fitting. The $R_{2\omega}^{H}$ vs. magnetic field angle $\varphi$ data is then fit as described in the main text. From the angle dependence fitting, the uncertainties in the coefficients of the $cos\varphi$ and $cos3\varphi$ components are obtained and combined to reflect the uncertainty in $R_{2\omega(ANE+AD)}^{H}$. The uncertainty of $R_{2\omega(ANE+AD)}^{H}$ is carried into a second linear regression fitting of $R_{2\omega(ANE+AD)}^{H}$ into inverse of total magnetic field, 1/(*B*_ext_+*B*_dem_-*B*_ani_). Then, the 95% confidence region involving slope and intercept is obtained from the fiting. Finally, the uncertainty of $R_{2\omega}^{\Delta T}$ is designated as the uncertainty in the intercept when the best fitted slope value is taken. On the other hand, the uncertainty of *R*_2ω_ is obtained from nonlinear least squares fitting of *R*_2ω_ vs. applied magnetic field. The uncertainty of *R*_USRMR_ is then simply calculated by combining the uncertainties of *R*_2ω_ and $R_{2\omega}^{\Delta T}$.


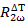


**Supplementary References**

^1^ Avci, C. O. et al., Nat Phys 11, 570–575 (2015).

^2^ Kikkawa, T. et al., Phys. Rev. B 92, 64413 (2015).
